# Molecular detection of Sodalis glossinidius, Spiroplasma and Wolbachia endosymbionts in wild population of tsetse flies collected in Cameroon, Chad and Nigeria

**DOI:** 10.21203/rs.3.rs-2902767/v1

**Published:** 2023-05-11

**Authors:** Youssouf Mouliom Mfopit, Judith Sophie Weber, Gloria Dada Chechet, Mahamat Alhadj Moussa Ibrahim, Djoukzoumka Signaboubo, Daniel Mbunkah Achukwi, Mohammed Mamman, Emmanuel Oluwadare Balogun, Mohammed Nasir Shuaibu, Junaidu Kabir, Soerge Kelm

**Affiliations:** Institut de Recherche Agricole pour le Développement; University of Bremen; Ahmadu Bello University; University of N'Djamena; Institut de Recherche en Elevage Pour Le Développement; TOZARD Research Laboratory; Ahmadu Bello University; Ahmadu Bello University; Ahmadu Bello University; Ahmadu Bello University; University of Bremen

**Keywords:** Tsetse flies, Symbionts, Wolbachia, Spiroplasma, Sodalis glossinidius, Cameroon, Chad, Nigeria

## Abstract

**Background:**

Tsetse flies are cyclical vectors of African trypanosomiasis. They have established symbiotic associations with different bacteria, which influence certain aspects of their physiology. The vector competence of tsetse flies for different trypanosome species is highly variable and is suggested to be affected by various factors, amongst which are bacterial endosymbionts. Symbiotic interactions may provide an avenue for the disease control. The current study provided the prevalence of 3 tsetse symbionts in *Glossina* species from Cameroon, Chad and Nigeria.

**Results:**

Tsetse flies were collected from five different locations and dissected. DNA was extracted and polymerase chain reaction PCR was used to detect the presence of *Sodalis glossinidius, Spiroplasma* sp and *Wolbachia* using specific primers. A total of 848 tsetse samples were analysed: *Glossina morsitans submorsitans* (47.52%), *Glossina palpalis palpalis* (37.26%), *Glossina fuscipes fuscipes* (9.08%) and *Glossina tachinoides* (6.13%). Only 95 (11.20%) were infected with at least one of the 3 symbionts. Among the infected, 6 (6.31%) were carrying mixed infection (*Wolbachia* and *Spiroplasma*). The overall symbiont prevalence was 0.88%, 3.66% and 11.00% respectively, for *Sodalis, Spiroplasma* and *Wolbachia*. Prevalence varied between countries and tsetse species. No *Spiroplasma* was detected in samples from Cameroon and no *Sodalis* was found in samples from Nigeria.

**Conclusion:**

The present study revealed for the first time, the presence of infection by *Spiroplasma* in tsetse in Chad and Nigeria. These findings provide useful information to the repertoire of bacterial flora of tsetse flies and incite to more investigations to understand their implication in the vector competence of tsetse flies.

## Background

Trypanosomiasis is one of the major endemic diseases in sub-Saharan Africa. It is caused by trypanosomes, an extracellular flagellated protozoan parasite of the genus *Trypanosoma*. The disease exists in two forms: The human African trypanosomiasis or sleeping sickness and the animal African trypanosomiasis. In humans, the disease is caused by two subspecies of *Trypanosoma brucei: Trypanosoma brucei rhodesiense* responsible for the acute form of the disease in eastern and southern Africa, while *Trypanosoma brucei gambiense* is responsible for the chronic form of the disease in western and central Africa [1]. Approximately 56 million people are estimated to be at different levels of risk of contracting HAT and more than a surface of 1.18 million Km^2^ are still at risk of *T.b. gambiense* infection [2]. In 2019 there were 992 cases recorded in Africa [1]. The animal form of the disease, animal African trypanosomiasis (AAT) is caused by several species and subspecies of trypanosomes including *Trypanosoma congolense, Trypanosoma vivax, Trypanosoma simiae, Trypanosoma uniforme, Trypanosoma godfreyi, Trypanosoma brucei brucei* and Trypanosoma grayi. They cause pathogenic infections in cattle, sheep, goats, pigs, dogs, camels and horses [3-5]. The disease is one of the major constraints to agricultural development on the continent.

Trypanosomes are cyclically transmitted between different vertebrate hosts by tsetse flies (Diptera: Glossinidae). Thirty-one species and subspecies of tsetse flies have been described. They can be grouped into three groups or subgenera based on common characteristics and morphology due to bio-ecological and genetic similarities: the riverine *Palpalis,* the savannah *Morsitans* and the forest *Fusca* [6, 7]. They acquire trypanosomes when feeding on an infected mammalian host. The trypanosomes undergo a series of transformations and multiplication in their gut and give rise to infective forms which will be inoculated into a new host during the feeding [8].

Tsetse gut harbours a diversity of bacteria acquired from the environment or maternally transmitted [9]. Previous studies have shown that these bacterial populations vary considerably depending both on the tsetse species or sub-species and the geographic origin of the flies [10]. This microbial community influences several aspects of tsetse’s physiology, including nutrition, fecundity development and maturation of the innate immune system and vector competence [11, 12].

Tsetse flies have established long-term associations with four vertically transmitted endosymbiotic bacteria including *Wigglesworthia glossinidia, Sodalis glossinidius, Wolbachia sp,* and *Spiroplasma sp* that was recently established as the fourth tsetse symbiont in *G. fuscipes fuscipes, G. tachinoides,* and *G. palpalis palpalis* [13]. They show different types of relation with their host.

All tsetse flies house *Wiggleworthia glossinidia,* the primary and obligate endosymbiont. It resides intracellularly within the bacteriome in the anterior midgut and also found extracellularly in milk gland of the fly [11]. *Wiggleworthia glossinidia* provides dietary supplements absent from the fly’s vertebrate blood-restricted diet, supports larval development and contributes to maturation of the adult immune system [12, 14].

*Wolbachia* endosymbionts are obligatory intracellular bacteria belonging to the Order Rickettsiales. *Wolbachia* infects a broad range of arthropod and filarial nematode species and is probably the most prevalent endosymbiont found in insect germlines [11]. Within the genus *Wolbachia,* 17 supergroups (A to Q) are currently recognized based on sequences of the five conserved genes *fbpA, coxA, ftsZ, gatB, coxA,* and *hcpA* and the amino acid sequences of the four hypervariable regions of the *Wsp* protein [11, 15]. The majority of insect infections fall into supergroups A and B [16].

In tsetse, *Wolbachia* resides mainly in reproductive tissues and is maternally transmitted from generation to generation by trans-ovarian transmission. They are also transferred horizontally among arthropods. *Wolbachia* infection in the tsetse host results in a variety of reproductive abnormalities such as parthenogenesis, male killing, feminization and cytoplasmic incompatibility [16-18].

Cytoplasmic incompatibility results in embryonic mortality in the progeny derived from matings between insects with different *Wolbachia* infection status: when an infected male mates with an uninfected female or a female infected with a different strain of the bacterium [16]. The presence of this symbiont in the tsetse fly *Glossina morsitans* has been associated with the induction of cytoplasmic incompatibility [17]. This effect confers indirect reproductive advantages to the infected females and is considered as a potential vector control alternative.

Some studies have shown that *Wolbachia* infections limited mosquito-transmitted pathogens including dengue virus, chikungunya virus, *Plasmodium* parasites, yellow fever virus, Zika virus and filarial nematodes [19-22].

The tsetse’s secondary and facultative symbiont is the commensal *Sodalis glossinidius*. It is a gramnegative organism belonging to the Enterobacteriaceae family. It is widely spread in numerous tissues of the fly (midgut, fat body, milk gland, salivary glands and reproductive system) and can be found both intracellularly and extracellularly [23]. The *Sodalis* genome consists of one circular chromosome of 4.17 Mbp, three extrachromosomal plasmids designated pSG1, pSG2, and pSG3, as well as a phage, ФSG1. However, its genome sequence shows reduced coding capacity with a large number of pseudogenes [24]. *Sodalis glossinidius* can be transmitted maternally via haemolymph, milk gland secretions, and horizontally during mating [11, 25]. In tsetse flies, the specific role of this symbiont is still not clear. However, it has been shown to affect host longevity and has been suspected to play a role in potentiating susceptibility to trypanosome infection in tsetse by influencing the efficacy of the tsetse immune system possibly through lectin-inhibitory activity [26].

The genus *Spiroplasma* belongs to the Mollicutes class, and the Tenericutes phylum. *Spiroplasma* are found abundantly in insects either in the gut or haemolymph where they have developed a large variety of interactions with the host that can be classified as commensal, pathogenic or mutualist [13].

It has been shown that *Spiroplasma* confers protection against pathogens e.g *Drosophila neotestacea* from a nematode [27], the pea aphid against fungi [28] and *Drosophila hydei* against a parasitoid wasp *Leptopilina heterotoma* [29]. However, reproductive alterations such as cytoplasmic incompatibility, malekilling and sex determination have been related to numerous species of *Spiroplasma* [13].

Recently, *Spiroplasma* has been established as a new class of tsetse symbiont in *G. fuscipes fuscipes, G. tachinoides,* and *G. palpalis palpalis*. The interactions between *Spiroplasma* and *Glossina* seem to be beneficial because of its ability to extend lifespan and reduce the vector competence for *Trypanosoma* [11, 30].

Current control measures against trypanosomiasis are mainly based on chemotherapy. In the absence of effective vaccine and to address the limitations associated with chemotherapy, disruption of trypanosomes transmission through vector control is crucial. Transmission of pathogens by vector depends on its vector competence, which can be affected by several factors, including vector endosymbionts [31]. Due to their importance, interactions between the symbionts and their hosts are being harnessed toward the development of novel approaches for vector and disease control [32-34].

The present study aims to investigate the presence of *Sodalis, Spiroplasma* and *Wolbachia* in wild population of tsetse flies from Cameroon, Nigeria and Chad.

## Results

For this study, 848 tsetse fly samples from three countries (Cameroon, Chad and Nigeria) were used. They belonged to four Glossina species (*Glossina morsitans submorsitans*: 403 (47.52%); *Glossina palpalis palpalis*: 316 (37.26%); *Glossina fuscipes fuscipes*: 77 (9.08%); *Glossina tachinoides*: 52 (6.13%)). Table 1 presents the distribution of the prevalence of different symbiotic bacteria.

Out of 848 flies, 95 (11.20%) were infected with at least one of the 3 endosymbionts. Among the infected, 6 (6.31%) were carrying mixed infection (only *Wolbachia-Spiroplasma*)

### Sodalis glossinidius infection prevalence

The presence of *Sodalis glossinidius* was investigated in 678 field-collected tsetse flies, originating from Cameroon (149), Chad (259) and Nigeria (270) using a *Sodalis Hemolysin* gene-based PCR. The *Sodalis* infection rates were 2.01% in Cameroon, 1.16% in Chad and 0.0% in Nigeria (Table 1). The prevalence of *S. glossinidius* infection did not differ significantly between Cameroon and Chad, while it was null in Nigeria.

**Figure 1:**
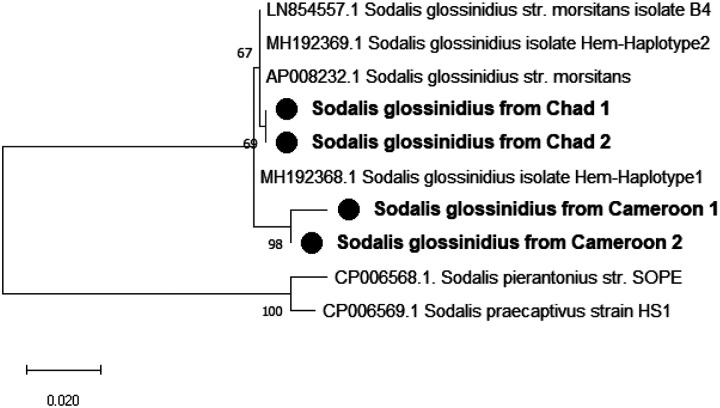
Phylogenetic tree of detected *Sodalis glossinidius’ hemolysin*partial gene and its closed relatives. The evolutionary history conducted in MEGA X, was inferred by using the Maximum Likelihood method and Hasegawa-Kishino-Yano model. This analysis involved 10 nucleotide sequences and a total of 596 positions in the final dataset. Our isolates are marked by a black circle. The percentage of replicate trees in which the associated taxa clustered together in the bootstrap test (1000 replicates) are shown next to the branches.

The sequence of our amplicons showed a high similarity (> 99%) with other *Sodalis’s Hemolysin* partial gene sequences found in NCBI database. The Maximum Likelihood phylogenetic tree (Fig. 1) showed that Cameroonian amplicons formed a different clade with amplicons from Chad.

### Spiroplasma infection prevalence

A total of 847 fly samples originated from Cameroon (154), Chad (423) and Nigeria (270) were used for the molecular detection of *Spiroplasma* using a 16S rRNA-based PCR approach with wspecF/wspecR primers. Only 31 samples were positive (3.66%). The prevalence varied significantly (p = 0.00) between countries (Table 1). *Spiroplasma* was detected in 0.0%, 0.95% and 10.00% of samples respectively from Cameroon, Chad and Nigeria.

**Figure 2:**
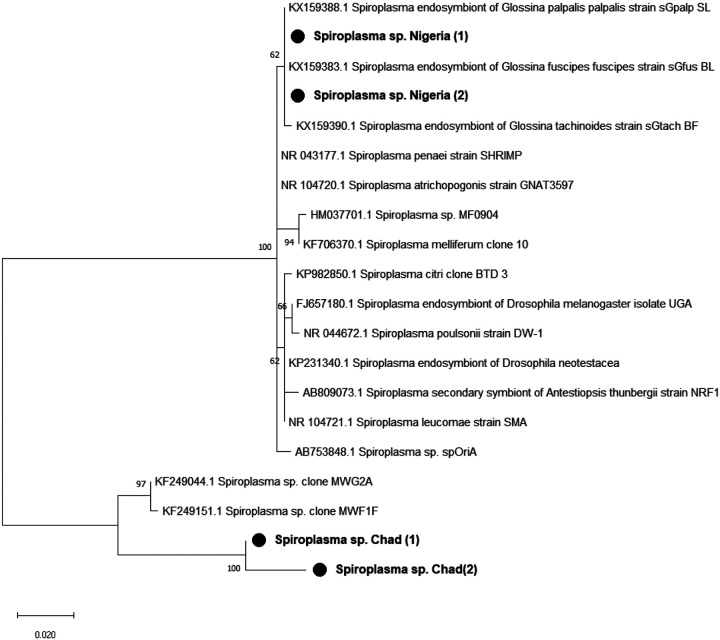
Phylogenetic tree of detected *Spiroplasma’s* 16S rRNA partial gene and its closed relatives. The evolutionary history conducted in MEGA X, was inferred by using the Maximum Likelihood method and Kimura 2-parameter model. This analysis involved 20 nucleotide sequences and a total of 383 positions in the final dataset. Our isolates are marked by a black circle. The percentage of replicate trees in which the associated taxa clustered together in the bootstrap test (1000 replicates) are shown next to the branches.

The sequence of our amplicons showed a high identity (> 99%) with other *Spiroplasma* 16S rRNA partial gene sequences found in NCBI database. The Maximum Likelihood phylogenetic tree (Fig. 2) showed that Chadian and Nigerian amplicons belong to 2 different clades.

### Wolbachia infection prevalence

A total of 582 tsetse samples (gut or abdomen) were screened for the presence of *Wolbachia* with a 16S rRNA-based PCR approach using the wspecF/wspecR set of primers. The samples were collected in 3 countries (Cameroon: 155, Chad: 157 and Nigeria: 270). The results of the screening (Table 1) showed that the prevalence of *Wolbachia* infections varied, but not significantly (p = 0.06) between the three countries. The *Wolbachia* prevalence in Cameroon, Chad and Nigeria was 9.68%, 7.00% and 14.07% respectively.

**Figure 3:**
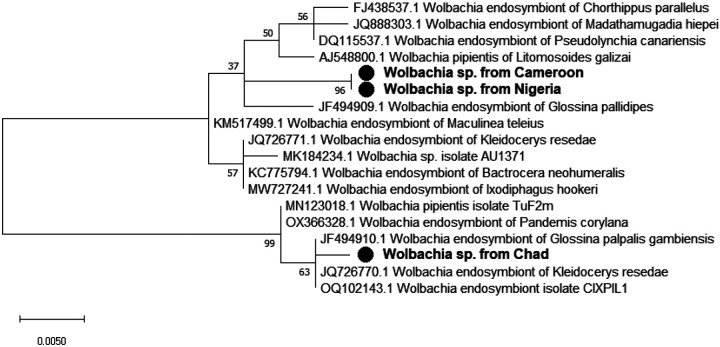
Phylogenetic tree of detected *Wolbachia’s* partial 16S rRNA gene and its closed relatives. The evolutionary history conducted in MEGA X, was inferred by using the Maximum Likelihood method and Kimura 2-parameter model. A discrete Gamma distribution was used to model evolutionary rate differences among sites. This analysis involved 18 nucleotide sequences and a total of 377 positions in the final dataset. Our isolates are marked by a black circle. The percentage of replicate trees in which the associated taxa clustered together in the bootstrap test (1000 replicates) are shown next to the branches.

The sequence of our amplicons showed a high identity (> 99%) with other *Wolbachia* 16S rRNA partial gene sequences found in NCBI database. The Maximum Likelihood phylogenetic tree (Fig. 3) showed that Cameroonian and Nigerian amplicons clustered together while they are far away from that from Chad.

All 16S rRNA and hemolysin gene sequences generated in this study have been deposited into GenBank under accession numbers OQ448931 to OQ448937 and OQ458709 to OQ458712.

## Prevalence according to the fly species

All the 4 species were found to be infected at different rates by the three symbiotic bacteria except *G. tachinoides* flies that were not infected by *S. glossinidius* (Table 2). There was no significant difference (p = 0.28) between the prevalence of *Sodalis* among the four species. The prevalence of *Spiroplasma* was significantly higher (p = 0.005) in *G.p. palpalis* (6.35%) and *G. tachinoides* (5.77%) compared to *G.m. submorsitans* (1.49) and *G.f. fuscipes* (2.60%). For *Wolbachia,* the prevalence was significantly lower (p = 0.005) in *G.m. submorsitans* (4.79%) compared to other fly species.

When the collection site is considered (prevalence compared between different fly species living in the same environment), there was no significant difference in symbiont prevalence among the three tsetse species in Lac Iro. However, in Yankari collection site, the *Wolbachia* infection was significantly related (p = 0.017) to *G. tachinoides*.

## Discussion

Owing to their restricted source of meal, the vertebrate blood, tsetse flies are highly dependent on their microbial flora which provide them with complementary nutrients [23]. Symbiotic associations have been established with some bacteria. In recent years, endosymbionts have received increased attention because of their large distribution in several insects and their effects on host physiology. Symbiotic interactions in tsetse flies have been discussed for their implication in vector competence of the flies [11].

In this study, tsetse symbiotic bacteria (*Sodalis*, *Spiroplasma* and *Wolbachia*) were detected in 11.20% of the tsetse samples examined with varying prevalence within countries, collection sites and tsetse species. This overall symbiont infection rate is lower compared to that of other previous studies [35-37]. Varying levels of infection rates may be attributed to environmental factors (vegetation, humidity, temperature) encountered in different ecological settings and to the intrinsic characters of each tsetse species.

### Sodalis prevalence

The overall *S. glossinidius* infection rate of 0.88% obtained in the present study is lower than the 12.69% global prevalence of *Sodalis* in tsetse samples from 15 African countries [35]. However, in the same study, similar prevalence was obtained in some West African countries: 0.48% in Burkina Faso, 0.00% in Mali, Ghana and Senegal. The *Sodalis* prevalence in Chad (1.16%) was lower than the 9.0% previously reported in the same area [38]. The prevalence in Cameroon was also lower than the 37.2% previously reported in the neighbouring area [36]. The variation may be due to PCR screening approach. The primers used in the previous works (pSG2 primers) were targeting a portion of the extrachromosomal plasmid 2 while in this present work, the primers (Hem primers) were targeting the nuclear hemolysin gene. Comparing different sets of primers, it has been reported that the use of the hemolysin gene provided a more reliable assessment of prevalence than the pSG2 that was giving higher but non-consistent prevalence [39]. Moreover, tsetse flies screened in Cameroon for the present study were all *G.p. palpalis* while the previous study screened *G. tachinoides* and *G. m. submorsitans*. Up to date in Nigeria, there is no published data on *S. glossinidius* prevalence in tsetse flies.

The presence of *S. glossinidius* has been suspected to be involved in vector competence of tsetse fly. But the confirmation is still under debate. Numerous reports showed a positive association between *Sodalis* and trypanosome establishment in the midgut possibly through lectin-inhibitory activity involving the production of N-acetyl glucosamine [26, 38, 40]. However, other studies reported the absence of association between the presence of *Sodalis* and that of Trypanosome [35-37].

### Spiroplasma prevalence

The *Spiroplasma* general infection rate of 3.66% obtained in the present study is much lower than 17.17% and 44.5% reported respectively in Burkina Faso for *G. tachinoides* and in Uganda for *G. f. fuscipes* [30, 41]. The study in Uganda showed a high variation of prevalence across the 26 sampling sites ranging from 0–62% and was correlated with the geographic origin and the season of collection of *G. f. fuscipes* [30]. The prevalence in Nigeria (10.0%) was significantly higher (p = 0.00) compared to that of Cameroon (0.0%) and Chad (= 0.95%). Few investigations have been done on the prevalence of *Spiroplasma* in tsetse flies in Africa. Up to date, there is no existing data on tsetse *Spiroplasma* prevalence in the 3 countries where our samples were collected.

*Spiroplasma* is known to induce reproductive abnormalities and pathogen protective phenotypes in various arthropods hosts [11]. A negative correlation of *Spiroplasma* with trypanosome infection was found in *G. f. fuscipes,* indicating that *Spiroplasma* infections may have an important effect in fly’s resistance to infection with trypanosomes [30, 42]. In an experimental study conducted by [42] on *G. f. fuscipes,* it was discovered that *Spiroplasma* infection induced changes in sex–biased gene expression in the reproductive tissues, adepletion in the availability of nutrients in pregnant females resulting in delayed larval development, and compromised sperm fitness. These findings indicate that *Spiroplasma* could be exploited for reducing tsetse population size and therefore, the disease transmission [42].

### Wolbachia prevalence

The *Wolbachia* global prevalence of 11.00% and countries prevalence (Table 1) obtained in the present study are in the range of prevalence reported by [18]. All these values were lower than 80.5% and 78.9% reported in Zambia for *G. m. morsitans* and *G. pallidipes* respectively [37]. In Cameroon and Chad, the prevalence previously obtained in the same and neighbouring study sites (67.6% and 14.5% respectively) were much higher than those of the present study [36, 38]. The primers used in this study were targeting the *Wolbachia* specific 16S rRNA while the previous studies used primers targeting the *Wolbachia* surface protein (*wsp*) gene. The comparison of results generated by 16S rRNA and wsp primers by [43] did not find a significant difference between the 2 primers, even though the prevalence with 16S rRNA primers (65%) was higher than the prevalence with wsp primers (60.5%). The tsetse species were globally different amongst the two studies. The prevalence in Nigeria (14.07%) was relatively higher than those of the two other countries (Cameroon: 9.68% and Chad: 7.00%). However, up to date there is no published data on *Wolbachia* infection in tsetse flies in Nigeria.

*Wolbachia* has received increased interest in recent years because of its high rates of distribution in a wide range of arthropods and nematodes, its unique effects on host physiology and its potential in disease control. Concerning trypanosomiasis, there is no confirmed implication of *Wolbachia* in tsetse vector competence. Investigations on the tripartite association between tsetse fly, *Wolbachia* and trypanosomes reported contrasting results. When *in G. f. fuscipes,* it was found a negative association between *Wolbachia* and trypanosome [44] suggesting a prevention of trypanosome infections by the presence of *Wolbachia*; some experimental studies did not find an association between the presence of *Wolbachia* and the ability of tsetse flies to harbor trypanosomes [36, 37, 43].

## Tsetse species

*Spiroplasma* was found to infect the 4 *Glossina* species, but with a low infection on *G.m. submorsitans* and *G.f. fuscipes*. On the contrary, the investigations by [13] found *Spiroplasma* infection only in palpalis group and nothing in the morsitans and fusca groups attributing this result to their frequent infection with *Wolbachia* which may have led to the development of competitive exclusion with *Spiroplasma. Wolbachia* was detected in all the 4 species with varying prevalence within species. This variation agrees with findings from similar studies on tsetse symbionts. In the present study, *G. tachinoides* was found to be more infected with *Wolbachia* than the 3 other tsetse species. *Sodalis* was not found in *G. tachinoides*. However, *Sodalis* is found to be infecting *G. tachinoides* in other studies [35, 36].

## Conclusion

The present study revealed for the first time, the presence of infection by *Spiroplasma* in tsetse in Chad and Nigeria. The infection rates of *Sodalis, Spiroplasma* and *Wolbachia* varied between countries and collection sites probably because of environmental factors. Few tsetse flies harboured symbiont co-infections. These findings provide useful information to the repertoire of bacterial flora of tsetse flies and incite to more investigations to understand the implication of these symbiotic bacteria in the vector competence of tsetse flies. More data could help the development of environmentally friendly methods for both tsetse and trypanosomiasis control.

## Methods

### Study areas

**Figure 4:**
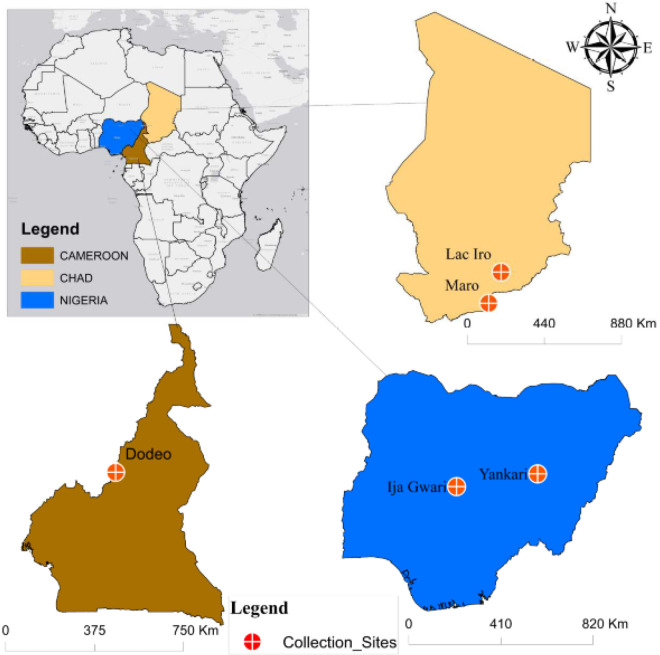
Study area. Tsetse flies were collected in Cameroon (Dodeo), in Chad (Maro and Lake Iro) and in Nigeria (Yankari Game reserve and in Ija-Gwari).

Tsetse flies used in this study were collected from three countries: Cameroon, Chad, Nigeria (Fig. 4).

In Cameroon flies were trapped in Dodeo (Latitude 7° 27.994’ N and Longitude 12° 04.101′ E) located in the “Faro et Déo” division of the Adamawa region. The village is close to the Cameroon-Nigeria Border. The vegetation is predominantly characterized by gallery forest along rivers and the climate type is of Sudano-Sahelian with two seasons: A rainy season from May to October and a dry season from November to April. This area has a dense hydrographic network and offers pastures for livestock [5, 36].

In Chad, flies were collected in Maro and Lake Iro areas, all situated in the Moyen-Chari province in southern Chad. The area has a climate of Sudano-Sahelian type with two seasons of equal duration. A rainy season from May to October and a dry season from November to April. Lac Iro is situated between the Latitude 9°59’ N and Longitude 19°26′ E, along the Salamat River. The vegetation is dominated by floodplains and dense forests containing shrubs. The area is considered as a buffer zone of the Zakouma national park where domestic and wild animals can meet [38]. Maro (8°24.807' N and 18°46.139' E) is located at the Chad-Central African Republic border. The area is crossed by many rivers and their multiple tributaries; the most important is the Chari River and its confluent the Grande Sido [45]. Maro is close to the Bamingui-Bangouran National Parc.

In Nigeria, flies were collected in Yankari and in Ija-Gwari. Yankari Game Reserve (9°45.240’N and 10°30.448E) is situated in the south-central part of Bauchi State in the North-East of the country. It covers an area of 2244 km^2^ and has a Sudan savannah vegetation. It has well-developed patches of woodland with scattered shrubs and trees. This national park has a rich wildlife diversity. Ija-Gwari is located at 9°18.860’N and 7° 26.814’E in Tafa Local Government Area of Niger State. The vegetation is of riverine fringing forest forming a dense two-storey canopy. The collection site is an open area where human activities (agriculture, cattle grazing) are regular [46].

### Sampling of tsetse

Biconical traps were used for this study. They were set up in various tsetse fly-favourable biotopes at distances of at least 100 m intervals. Tsetse flies were collected once a day and transported in cool boxes to the base camp. The collected flies were identified using morphological identification keys [47, 48], numbered and dissected on the same day.

### Dissection

Flies were dissected in a drop of sterile saline solution as described by [5, 46]. The wings were removed first, followed by legs, proboscis, salivary glands and gut. After each fly, dissection tools (forceps, pins and slide) were decontaminated by immersion in 5% sodium hypochlorite for approximately 10 minutes, followed by immersion in 70% ethanol and final immersion in sterile normal saline.

Wings were stored dry for geometric morphometrics. Legs, proboscis and salivary glands were preserved in nucleic acid preservation agent (NAPA: 25 mM sodium citrate, 10 mM EDTA, 70 g ammonium sulphate/100 mL solution, pH 7.5) in 1.5 mL cryotubes. Gut tissue was homogenised in 200 μL of 50 mM Tris-Cl, pH 9.0, using four 2.38 mm metal beads (MoBio Laboratories, Carlsbad, CA, USA). Fifty microliters of the homogenate were added to 500 μL of NAPA.

Most of the flies from Chad were dead. For this reason, after the removal of wings, legs and proboscis, the entire abdomen was transferred in ethanol.

### DNA extraction

DNA was extracted from homogenised gut tissue in NAPA with the DNeasy Blood and Tissue Kit (Qiagen, Germany) according to the manufacturer’s instructions with slight adjustments (100 μL of homogenate were used for purification and 100 μL elution buffer were used at elution step).

For the dead flies from Chad, DNA was extracted from the abdomen using 5% Chelex-100 Resin (BIO-RAD, Hercules, California, USA). Briefly, the abdomen was transferred from ethanol to a new 1.5 mL centrifuge tube and allowed to dry. The abdomen was then crushed using the micro-pipette tips. Thereafter, 100 μL of chelex 5% solution were added. The mixture was vortexed and incubated at 56° C for 30 min in a Thermomixer. After this incubation, the mixture was vortexed and incubated for an additional 5 min at 95° C. The mixture was then mixed thoroughly before brief centrifugation at 7000 rpm for 1 min and stored at −20°C.

#### Molecular detection of Wolbachia

The detection of *Wolbachia* was carried out by amplifying a 438 bp fragment of the 16S rRNA gene with the 16S W-Spec primers (Table 3) designed by [49].

PCR amplifications were performed in 20 μL reaction mixture containing 1 X DreamTaq buffer (10X), 150 μM dNTPs, 0.2 μM of each primer, 0.5 U of Dream Taq polymerase and 2 μl of template DNA. The cycling condition was as described by. It started by 95°C for 5 min followed by 35 cycles of 95°C for 30 s, 30 s at 54°C, 1 min at 72°C and a final extension step of 72°C for 10 min. After amplification, the PCR products were analyzed by electrophoresis on a 1.5% agarose gel containing Stain-G and visualized under UV light.

#### Molecular detection of S. glossinidius

The presence of *S. glossinidius* was determined by PCR with Hem primers (Table 3) that target the gene encoding the haemolysin protein of the bacterium [50]. The primers targeted a 650 bp fragment of the hemolysin gene. The PCR reaction was performed in a final volume of 20 μL containing 1X Dream taq buffer, 150 μM dNTPs, 0.2 μM of each primer, 0.5 U of Dream Taq polymerase and 2 μL of DNA template. PCR cycles were: 95°C for 5 min; 35 cycles of 95°C for 30 s, 54°C for 30 s and 72°C for 60 s; and a final elongation at 72°C for 10 min.

#### Molecular detection of Spiroplasma

The screening for *Spiroplasma* was carried out by amplifying the 455 bp fragment of the 16S rRNA as described by [13] with specific primers (Table 3).

PCR reactions were performed in 20 μl reaction mixture containing 1X DreamTaq buffer, 150 μM dNTPs, 0.65 μM of each primer, 0.5 U of DreamTaq polymerase and 2 μl of template DNA. The PCR temperature profile was 95°C for 5 min followed by 35 cycles of 95°C for 30 s, 30 s at 59°C, 1 min at 72°C and a final extension step of 72°C for 10 min.

### Sequencing

Selected positive samples, were re-amplified in PCR reaction volume of 50 μL (maintaining the same concentration of reactants). Five microliters were visualised on 1.5% agarose gel for confirmation of the amplification. The remaining 45 μL PCR products were separated on 2% agarose gel. The amplicons bands were extracted and purified using GeneJET Gel Extraction Kit (Thermo Scientific) according to manufacturer instructions. The purified amplicons were sent for sanger sequencing to a commercial company (SeqLab, Göttingen, Germany).

### Phylogenetic analysis

Obtained sequences were manually checked and edited using Geneious Pro version 5.5.9 software. The Basic Local Alignment Search Tool (BLASTn) from National Center for Biotechnology Information (NCBI) was used to confirmed the identity of the amplicons and to determine the closest related sequences in the GenBank.

Sample sequences and reference sequences obtained from NCBI were aligned using MUSCLE alignment tool with its default setting implemented in MEGAX [51] which was also used to infer phylogenetic relationships. Maximum likelihood method was performed with the Kimura-2 model [52] for *Wolbachia* and *Spiroplasma*, then Hasegawa-Kishino-Yano model [53] for *Sodalis* as determined by the MEGA model finder tool with 1000 bootstraps replicates.

### Statistics Data analysis

Endosymbionts hosted by tsetse flies were expressed in percentage as symbiont prevalence. The Pearson’s chi-square test (χ^2^) was used to compare symbiont prevalences between countries and collection sites. The differences were considered significant when the p-values were lower than 0.05. Statistical tests were performed using SPSS for Windows, version 20.

## Figures and Tables

**Table 1:** Distribution of endosymbiont infection in different study areas

Countries	Sites	Species	*Sodalis*	*Spiroplasma*	*Wolbachia*
**Cameroon**	Dodeo	*G.p. palpalis*	3/149 (2.01%)	0/154 (0.0%)	15/155 (9.68%)
			**3/149 (2.01%)**	**0/154 (0.0%)**	**15/155 (9.68%)**
**Chad**	Maro	*G.f. fuscipes*	2/69 (2.90%)	2/69 (2.90%)	8/69 (11.59%)
	Lac Iro	*G.f. fuscipes*	0/6 (0.0%)	0/8 (0.0%)	0/2 (0.0%)
		*G.m. submorsitans*	1/182 (0.55%)	2/343 (0.58%)	3/86 (3.49%)
		*G. tachinoides*	0/2 (0.0%)	0/3 (0.0%)	
			**3/259 (1.16%)**	**4/423 (0.95%)**	**11/157 (7.00%)**
**Nigeria**	Ija-Gwari	*G.p. palpalis*	0/161 (0.0%)	20/161 (1.24%)	23/161 (14.29%)
	Yankari	*G.m. submorsitans*	0/60 (0.0%)	4/60 (6.66%)	4/60 (6.67%)
		*G. tachinoides*	0/49 (0.0%)	3/49 (6.12%)	11/49 (22.45%)
			**0/270 (0.0%)**	**27/270 (10.0%)**	**38/270 (14.07%)**
	**Total**	**6/678 (0.88%)**	**31/847 (3.66%)**	**64/582 (11.00%)**

**Table 2:** Distribution of endosymbiont infection per fly species

	Sodalis	Spiroplasma	Wolbachia
G.f. fuscipes	2/75 (2.67%)	2/77 (2.60%)	8/71 (11.27%)
G.m. submorsitans	1/242 (0.41%)	6/403 (1.49%)	7/146 (4.79%)
G.p. palpalis	3/310 (0.97%)	20/315 (6.35%)	38/316 (12.02%)
G. tachinoides	0/51 (0.0%)	3/52 (5.77%)	11/49 (22.45%)
p-value (Pearson χ^2^)	**0.283**	**0.005**	**0.005**

**Table 3:** List of primers used in this study

Organisms	Primer name	Nucleotide sequences (5’ – 3’)	Amplicon size (bp)	References
*Sodalis glossinidius*	HemF	ATGGGAAACAAACCATTAGCCA	650	[50]
	HemR	TCAAGTGACAAACAGATAAATC		
*Spiroplasma*	63F	GCCTAATACATGCAAGTCGAAC	455	[13]
	TKSSsp	TAGCCGTGGCTTTCTGGTAA		
*Wolbachia*	WspecF	CATACCTATTCGAAGGGATAG	438	[49]
	WspecR	AGCTTCGAGTGAAACCAATTC		

## Abbreviations

BLAST: Basic Local Alignment Search Tool
NAPA: Nucleic Acid Preservation Agent
NCBI: National Center for Biotechnology Information
